# Acute heart failure with mildly reduced ejection fraction and myocardial infarction: a multi-institutional cohort study

**DOI:** 10.1186/s12872-023-03286-9

**Published:** 2023-05-23

**Authors:** Ming-Shyan Lin, Po-Chang Wang, Meng-Hung Lin, Ting-Yu Kuo, Yu-Sheng Lin, Tien-Hsing Chen, Ming-Horng Tsai, Yao-Hsu Yang, Chun-Liang Lin, Chang-Min Chung, Pao-Hsien Chu

**Affiliations:** 1 Division of Cardiology, Department of Internal Medicine, Chang Gung Memorial Hospital Chiayi Branch, Chiayi, Taiwan; 2 Graduate Institute of Clinical Medical Sciences, College of Medicine, Chang Gung University, Taoyuan, Taiwan; 3 Department of Nursing, Chang Gung University of Science and Technology, Chiayi, Taiwan; 4 Health Information and Epidemiology Laboratory, Chang Gung Memorial Hospital Chiayi Branch, Chiayi, Taiwan; 5 Division of Cardiology, Department of Internal Medicine, Chang Gung Memorial Hospital Chiayi Branch, Keelung, Taiwan; 6 Department of Pediatrics, Chang Gung Memorial Hospital, Yunlin, Taiwan; 7 Department of Traditional Chinese Medicine, Chang Gung Memorial Hospital Chiayi Branch, Chiayi, Taiwan; 8 School of Traditional Chinese Medicine, College of Medicine, Chang Gung University, Taoyuan, Taiwan; 9 Department of Nephrology, Chang Gung Memorial Hospital Chiayi Branch, Chiayi, Taiwan; 10 Division of Cardiology, Department of Internal Medicine, Chang Gung Memorial Hospital Linkou Branch, No.5, Fu-Hsing Street, Gueishan District, Taoyuan, 33305 Taiwan; 11 Institute of Stem Cell and Translational Cancer Research, Chang Gung Memorial Hospital Chang Gung University College of Medicine, Taipei, Taiwan

**Keywords:** Heart failure mildly reduced ejection fraction, Myocardial infarction, Mortality

## Abstract

**Background:**

Little research has been done on ischemic outcomes related to left ventricular ejection fraction (EF) in acute decompensated heart failure (ADHF).

**Methods:**

A retrospective cohort study was conducted between 2001 and 2021 using the Chang Gung Research Database. ADHF Patients discharged from hospitals between January 1, 2005, and December 31, 2019. Cardiovascular (CV) mortality and heart failure (HF) rehospitalization are the primary outcome components, along with all-cause mortality, acute myocardial infarction (AMI) and stroke.

**Results:**

A total of 12,852 ADHF patients were identified, of whom 2,222 (17.3%) had HFmrEF, the mean (SD) age was 68.5 (14.6) years, and 1,327 (59.7%) were males. In comparison with HFrEF and HFpEF patients, HFmrEF patients had a significant phenotype comorbid with diabetes, dyslipidemia, and ischemic heart disease. Patients with HFmrEF were more likely to experience renal failure, dialysis, and replacement. Both HFmrEF and HFrEF had similar rates of cardioversion and coronary interventions. There was an intermediate clinical outcome between HFpEF and HFrEF, but HFmrEF had the highest rate of AMI (HFpEF, 9.3%; HFmrEF, 13.6%; HFrEF, 9.9%). The AMI rates in HFmrEF were higher than those in HFpEF (AHR, 1.15; 95% Confidence Interval, 0.99 to 1.32) but not in HFrEF (AHR, 0.99; 95% Confidence Interval, 0.87 to 1.13).

**Conclusion:**

Acute decompression in patients with HFmrEF increases the risk of myocardial infarction. The relationship between HFmrEF and ischemic cardiomyopathy, as well as optimal anti-ischemic treatment, requires further research on a large scale.

**Supplementary Information:**

The online version contains supplementary material available at 10.1186/s12872-023-03286-9.

## Introduction

Globally, 64 million people suffer from heart failure (HF), with a prevalence of 1–3% in adults and more than 6% in Taiwan [1]. Half of acute decompensated heart failure (ADHF) patients died within five years while ischemic heart disease (IHD) is associated with poorer prognoses, insurance burden, and worsening mortality [2]. IHD was more common in Southeast Asia, and the Western Pacific region (more than 50% in ADHF) in REPORT-HF registry [2, 3]. Even with the complexity of etiology in ADHF, left ventricular ejection fraction (LVEF) can be a simple tool for phenotyping in many randomized controlled trials and observative studies [4]. HFmrEF is an intermediate phenotype between HFrEF and HFpEF sharing heterogenous outcomes [1, 3–5]. The HFmrEF is similar to the HFrEF on mortality and HF rehospitalization, but it is also similar to the HFpEF on comorbidities [6–11]. The use of guideline-directed medical therapy (GDMT) [4, 12] confirmed a survival effect on chronically stable HFrEF while the PARAGON-HF [13] and DELIVER trials [14] had conflicting outcomes in patients with EF > 40%, particularly when it came to ADHF.

As with HFrEF, HFmrEF was associated with a significantly higher incidence of IHD than HFpEF [15], and established IHD had a considerably poorer prognosis [6, 8, 9, 16, 17]. Sudden cardiac death may be more prevalent among patients with HFrEF and HFmrEF due to underlying coronary events [18]. The results of the ESC HF LT Registry indicate that myocardial ischemia contributes to hospitalization in patients with HFmrEF [19]. As the EF decreased by 45% in the CHARM study, the risk of myocardial infarction increased linearly [20]. Further, Vedin O and colleagues demonstrated that HFmrEF was associated with similar MI risks as HFrEF, as well as higher risks than HFpEF [2, 9]. After acute compensation, sequential MI may increase in HFmrEF with potential IHD risk; however, it is relevant to note that most previous studies lacked adequate propensity matching, short-term follow-up, mixed with chronic or acute HF, and a diverse distribution of EF categories. A comprehensive cardiovascular outcome study and long-term observation of ADHF were therefore needed beyond heart failure and cardiovascular mortality.

To resolve the conflict in HFmrEF outcomes, we compared three cohorts by ejection fraction and balanced covariables through propensity matching. Furthermore, ischemic events including subsequent myocardial infarction and stroke after discharge were also surveyed using long-term and large-scale observation. Our goal was to compare admission characteristics, in-hospital course, discharge medication, and cause-specific post-discharge outcomes for patients with acute HFpEF, HFmrEF, and HFrEF.

## Methods

### Database

A total of 10,000 beds are available at Taiwan’s CGMH Medical System, which includes four tertiary care hospitals and three major teaching hospitals. The CGMH medical database contains records from 1 to 2001 to 31 March 2021, including diagnoses, laboratory data, medications, echocardiography, imaging, and detailed charts. In spite of the fact that the patient’s identity (i.e. the chart number or national identification number) was encrypted, every patient was assigned a personal identification number (PIN), which could be linked to their medical records. The CGMH medical database is described in greater detail elsewhere [21]. Research protocol approved by CGMH Institutional Review Board (IRB no. 202100393B0C601).

### Study design

**Fig. 1:**
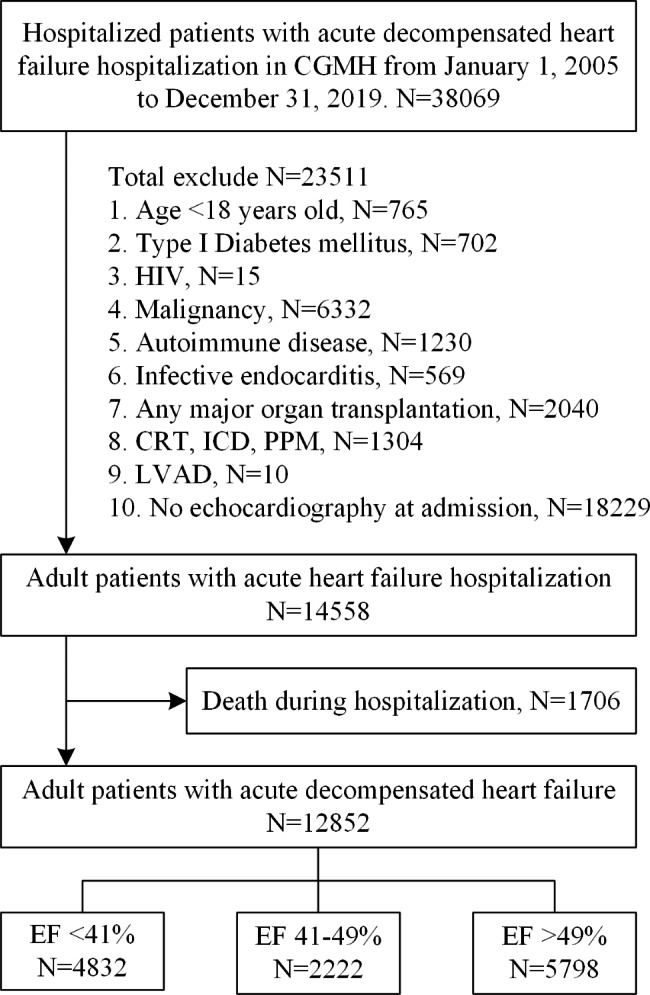
Enrollment and flowchart of the study

The retrospective, multicenter cohort study examined 38,069 hospitalized patients with acute decompensated heart failure who were discharged during the period of January 1, 2005, through December 31, 2019. It excludes individuals under the age of 18, type I diabetes mellitus, HIV, malignancy, autoimmune diseases, infective endocarditis, major organ transplants, cardiac resynchronization therapy devices, cardioverter defibrillators (ICD), permanent pacemakers (PPM), and absence of in-hospital echocardiography. The echographic examination and the ESC/AHA guidelines definition [4, 12] was used to classify 12,852 patients into three categories: HFrEF, HFmrEF, and HFpEF (Fig. 1).

### Baseline characteristics

There were a number of HF-related comorbidities present, including hypertension, diabetes, dyslipidemia, atrial fibrillation, peripheral arterial disease, venous thromboembolism, chronic obstructive pulmonary disease, gouty arthritis, gastrointestinal bleeding, intracranial hemorrhage, ischemic heart disease, old ischemic stroke, status following coronary artery bypass surgery, and valve surgery. At least once in the one-year period before discharge, comorbidities were identified from outpatient, emergency, or inpatient records.

### Discharge medications

A review of medications prescribed within six months of discharge was conducted. The drugs prescribed included antiplatelets, oral anticoagulants, renin-angiotensin system inhibitors (RASi), beta-blockers, dihydropyridine calcium channel blockers (DCCB), calcium channel blockers (CCB), mineralocorticoid receptor antagonists (MRA), diuretics, oral glycemic agents (OHA), insulin therapy, and Satin. In accordance with World Health Organization Anatomical Therapeutic Chemical codes and Taiwan National Health Insurance reimbursement codes, guidelines-directed medical therapy (GDMT) includes RASi, beta-blockers, and MRA.

### Survival and cardiovascular outcomes

The primary outcomes are cardiovascular death and rehospitalization for heart failure. As well as the primary outcomes of all-cause mortality, individual HF rehospitalizations, CV death, acute myocardial infarction (AMI), and ischemic stroke (IS), secondary outcomes are also evaluated. According to ICD-9-CM and ICD-10-CM diagnostic codes for any inpatient diagnosis (eAppendix 1 in the Additional File), the occurrence of cardiovascular death and heart failure rehospitalization was calculated. As defined by the National Registry of Death, CV death includes death from heart disease, hypertension, and cerebrovascular disease. Among the principal discharge diagnoses of hospitalization were ischemic stroke, acute myocardial infarction, and heart failure rehospitalization. From the index date to the date of an event or death, or March 31, 2021, whichever occurred first, each patient was followed.

### Statistical analysis

The characteristics of patients with each HFrEF, HFmrEF, and HFpEF grade were compared in this study. A Chi-Squared test was used to compare categorical variables between HFrEF, HFmrEF, and HFpEF. ANOVA was used to compare continuous variables between HFrEF, HFmrEF, and HFpEF. Over a 5-year follow-up period, Kaplan–Meier plots were constructed comparing the primary outcome, all-cause mortality, CV death, heart failure rehospitalization, AMI and ischemic stroke. Events are defined as the occurrence of the outcomes of interest within five years of discharge. For survival curve analysis, patients with no events and no deaths during follow-up were censored. A Cox proportional hazard model was used to compare the risks of fatal outcomes (all-cause and cardiovascular death). In comparing non-fatal outcomes (HF rehospitalization, AMI, and ischemic stroke) between groups, Fine and Gray subdistribution hazard models were used. SAS version 9.4 (SAS Institute, Cary, NC) was used for all statistical analyses.

## Results

### Baseline characteristic of ADHF among three subgroups

Table 1 summarizes the baseline clinical characteristics of the patients. The total number of subjects enrolled was 12,852 (HFrEF 37.6%, HFmrEF 17.3%, HFpEF 45.1%), with a mean in-hospital mortality of 11.7% (13.8% in HFrEF, 10.3% in HFmrEF, 10.5% in HFpEF) (eTable 1 and eFigure 1 in the Additional File). HFrEF was younger than HFpEF and HFmrEF and had a higher rate of ischemic heart disease and post-CABG. In addition to severe pulmonary and hepatic congestion, HFrEF had significantly higher levels of ALT, AST, and NT-proBNP. As with HFmrEF, HFpEF patients had multicomorbidity, including AF and advanced CKD. The HFrEF had a higher proportion of RASi, BBs, and MRA prescriptions before discharge than the HFpEF and HFmrEF, who also had more antiplatelet agents, OAC, and statins.

**Table 1:** Baseline characteristics of the study patients

Variable	HFrEF (N, %)		HFmrEF (N, %)		HFpEF (N, %)		*P-value*
Total	4832		2222		5798		
Age, mean (SD), years	65.0	(16.1)	68.5	(14.6)	72.6	(13.6)	< 0.001
Age group, years							< 0.001
18–64	2298	(47.6)	842	(37.9)	1487	(25.7)	
65–74	980	(20.3)	536	(24.1)	1363	(23.5)	
≥ 75	1554	(32.2)	844	(38.0)	2948	(50.9)	
Sex							< 0.001
Male	3284	(68.0)	1327	(59.7)	2623	(45.2)	
Female	1548	(32.0)	895	(40.3)	3175	(54.8)	
HF diagnosis year							< 0.001
Before 2016	3477	(72.0)	1749	(78.7)	4276	(73.8)	
After 2016	1355	(28.0)	473	(21.3)	1522	(26.3)	
Hospital level							< 0.001
Medical center	3338	(69.1)	1627	(73.2)	3927	(67.7)	
Non-medical center	1494	(30.9)	595	(26.8)	1871	(32.3)	
Comorbidities							
Hypertension	3052	(63.2)	1598	(71.9)	4382	(75.6)	< 0.001
Diabetes mellitus	2011	(41.6)	1043	(46.9)	2563	(44.2)	< 0.001
Dyslipidemia	1445	(29.9)	806	(36.3)	1969	(34.0)	< 0.001
Atrial fibrillation	1330	(27.5)	657	(29.6)	1997	(34.4)	< 0.001
Peripheral arterial disease	249	(5.2)	127	(5.7)	329	(5.7)	0.437
VTE	160	(3.3)	80	(3.6)	327	(5.6)	< 0.001
COPD	722	(14.9)	404	(18.2)	1250	(21.6)	< 0.001
Gouty arthritis	603	(12.5)	277	(12.5)	840	(14.5)	0.004
Gastrointestinal bleeding	951	(19.7)	544	(24.5)	1551	(26.8)	< 0.001
Intra-cranial hemorrage	77	(1.6)	40	(1.8)	115	(2.0)	0.323
Ischemic heart disease	2946	(61.0)	1503	(67.6)	2775	(47.9)	< 0.001
Old ischemic stroke	585	(12.1)	326	(14.7)	993	(17.1)	< 0.001
S/p CABG	304	(6.3)	143	(6.4)	214	(3.7)	< 0.001
Valve replacement	115	(2.4)	80	(3.6)	289	(5.0)	< 0.001
Laboratory data, mean (SD)							
Hemoglobin, g/dL	12.1	(2.5)	11.3	(2.4)	10.9	(2.3)	< 0.001
Creatinine, mg/dL	1.9	(2.1)	2.4	(2.8)	2.0	(2.2)	< 0.001
Uric acid, mg/dL	8.4	(2.8)	7.6	(2.8)	7.5	(2.6)	< 0.001
ALT, U/L	138.6	(532.9)	79.3	(278.3)	55.0	(210.0)	< 0.001
AST, U/L	179.3	(857.6)	111.7	(451.2)	76.3	(415.8)	< 0.001
NT-ProBNP, pg/mL	8307.8	(10527.6)	5734.7	(5211.3)	5820.6	(7398.5)	0.473
BNP, pg/mL	1452.3	(1540.0)	1370.6	(1452.0)	905.0	(1050.7)	< 0.001
eGFR, mL/min/1.73m^2^							< 0.001
≥ 60	2699	(58.8)	1153	(55.6)	3036	(56.8)	
30–59	1052	(22.9)	439	(21.2)	1125	(21.0)	
16–30	345	(7.5)	133	(6.4)	466	(8.7)	
Dialysis	493	(10.7)	348	(16.8)	721	(13.5)	
Hospitalized Intervention							
Intubation/ventilation	423	(8.8)	194	(8.7)	370	(6.4)	< 0.001
Intensive Care Unit stay	1617	(33.5)	769	(34.6)	1478	(25.5)	< 0.001
Intensive Care Unit stay, mean (SD), days	7.1	(9.0)	6.1	(7.6)	6.6	(9.0)	0.018
NIPPV	118	(2.4)	47	(2.1)	133	(2.3)	0.689
CPCR	17	(0.4)	3	(0.1)	11	(0.2)	0.126
Cardioversion	43	(0.9)	19	(0.9)	29	(0.5)	0.038
Blood transfusion	185	(3.8)	118	(5.3)	449	(7.7)	< 0.001
Hemodialysis	96	(2.0)	81	(3.7)	113	(2.0)	< 0.001
Coronary PCI with stenting	76	(1.6)	31	(1.4)	43	(0.7)	< 0.001
Discharge medications							
Antiplatelet	2714	(56.2)	1397	(62.9)	2581	(44.5)	< 0.001
OAC	3271	(67.7)	1632	(73.5)	3447	(59.5)	< 0.001
RASi	3630	(75.1)	1534	(69.0)	3029	(52.2)	< 0.001
Beta-blockers	2965	(61.4)	1222	(55.0)	2160	(37.3)	< 0.001
DCCB	1236	(25.6)	598	(26.9)	1615	(27.9)	0.031
CCB	83	(1.7)	86	(3.9)	568	(9.8)	< 0.001
Diuretics	3395	(70.3)	1363	(61.3)	3463	(59.7)	< 0.001
MRA	1409	(29.2)	315	(14.2)	695	(12.0)	< 0.001
OHA	1042	(21.6)	529	(23.8)	1204	(20.8)	0.012
Insulin	208	(4.3)	147	(6.6)	349	(6.0)	< 0.001
Statin	1282	(26.5)	693	(31.2)	1228	(21.2)	< 0.001

Abbreviations: ALT, Alanine aminotransferase; AST, Aspartate aminotransferase; BNP, B-type natriuretic peptide; CCB, calcium channel blockers; COPD, Chronic obstructive pulmonary disease; CPCR, Cardio-Pulmonary-Cerebral-Resuscitation; DCCB, dihydropyridine calcium channel blockers; eGFR, estimated Glomerular filtration rate; HFmrEF, heart failure with mid-range ejection fraction; HFpEF, heart failure with preserved ejection fraction; HFrEF, heart failure with reduced ejection fraction; MRA, mineralocorticoid receptor antagonist; NIPPV, Noninvasive positive pressure ventilation; NT-ProBNP, N-terminal Pro-Brain Natriuretic Peptide; OAC, oral anticoagulants; OHA, oral hypoglycemic agent; PCI, percutaneous coronary intervention; RASi, angiotensin converting enzyme inhibitors, angiotensin receptor blocker, or angiotensin receptor–neprilysin inhibitor; S/p CABG, status post coronary artery bypass graft; VTE, Venous thromboembolism.

### Outcomes analysis by left ventricular ejection fraction

**Fig. 2:**
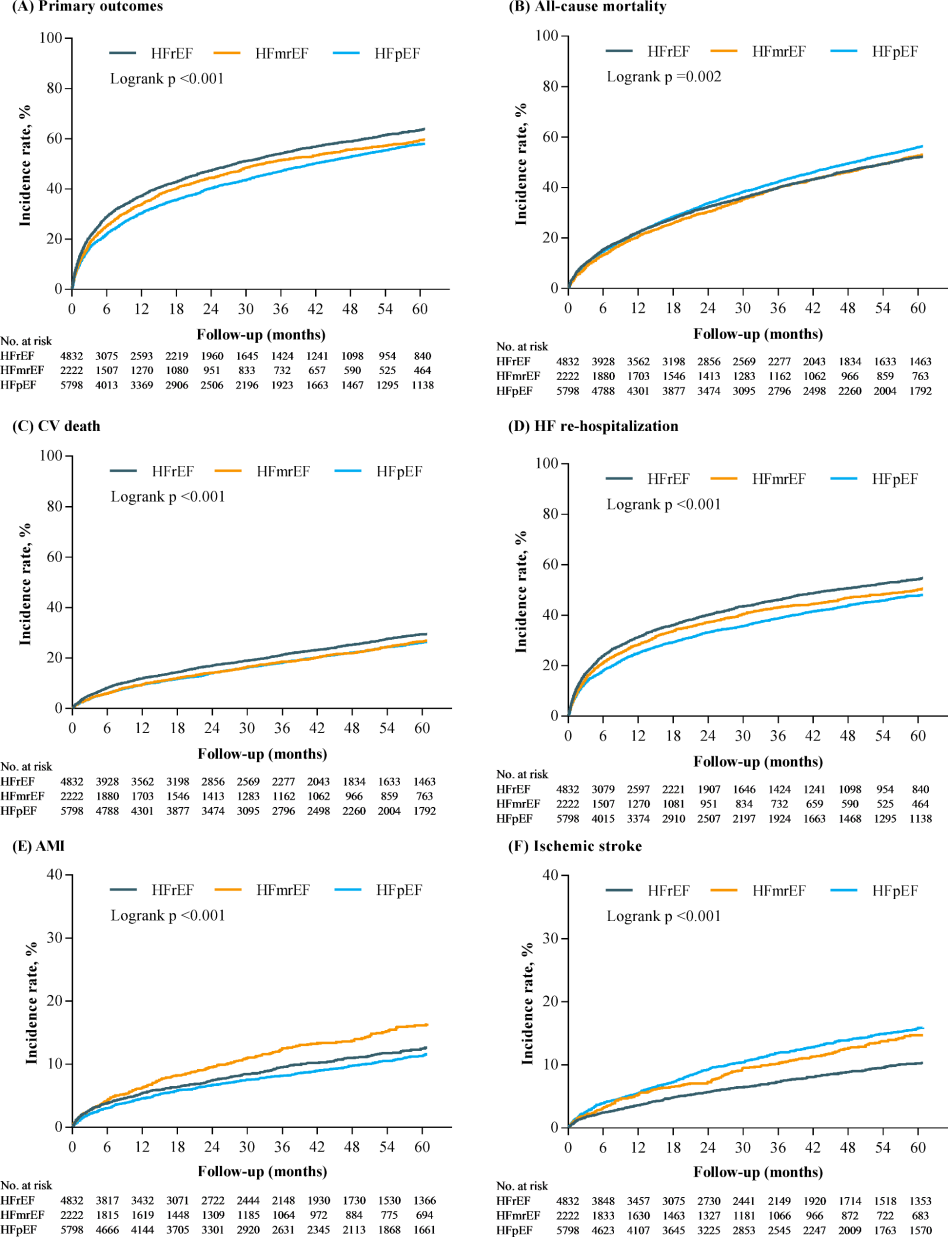
Kaplan–Meier curves for the primary outcome (A), all-cause mortality (B), CV death (C), HF rehospitalization (D), acute myocardial infarction (E), and ischemic stroke (F) of patients with decompensated heart failure by left ventricular ejection fraction

**Fig. 3:**
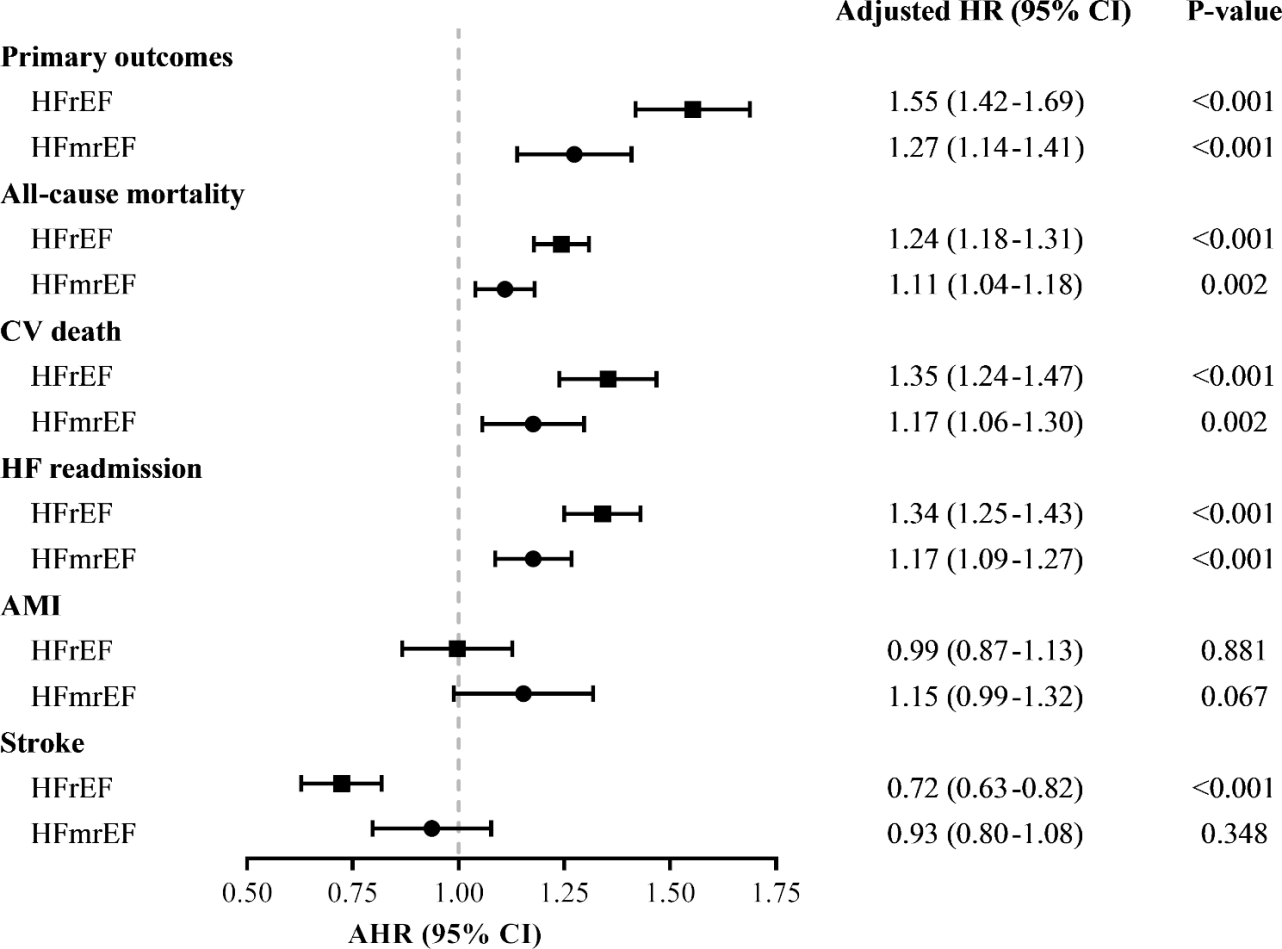
A comparison of the prognostic factors for outcomes based on left ventricular ejection fraction after adjustment

In the Table 2, all outcomes are analyzed according to the individual definition based on 2.8 years of follow-up (interquartile range, 0.9 to 5.8 years). HFrEF has a significantly higher incidence event of primary endpoint (HFrEF 58.2%; AHR, 1.55; 95% CI, 1.42 to 1.69; HFmrEF 56.4%; AHR, 1.27; 95% CI, 1.14 to 1.41; HFpEF 51.2%, respectively) (Fig. 2 and eTable 2). Additionally, HFrEF has the highest risk for individual outcomes on CV death (AHR, 1.35; 95% CI, 1.24 to 1.47; *P* < 0.001) and HF rehospitalization (AHR, 1.34; 95% CI, 1.25 to 1.43; *P* < 0.001) (Fig. 2 and eTable 2). HFpEF has a significantly higher proportion of all-cause mortality compared with the other two groups (HFrEF 56.8%; HFmrEF 61.3%; HFpEF 63.8%; *P* < 0.001) (Table 2). After adjustment, HFrEF (AHR, 1.24; 95% CI, 1.18 to 1.31; *P* < 0.001) and HFmrEF (AHR, 1.11; 95% CI, 1.04 to 1.18; P = 0.002) have significant risk compared to HFpEF (eTable 2 and Fig. 3). For AMI, HFmrEF is the highest risky group compared with others (HFrEF 9.9%; AHR, 0.99; 95% CI, 0.87 to 1.13; *P* = 0.881; HFmrEF 13.6%; AHR, 1.15; 95% CI, 0.99 to 1.32; *P* < 0.001; HFpEF 9.3%, respectively.); however, HFpEF has significant more events of sequent stroke than other two groups (HFrEF 7.8%; HFmrEF 11.2%; HFpEF 12.0%; *P* < 0.001). After adjustment, HFrEF still has a significantly lower risk than HFpEF (AHR, 0.72; 95% CI, 0.63 to 0.82; *P* < 0.001) (eTable 2 and Fig. 3). Despite all-cause mortality, all outcomes initially had similar trends and differences during the first 6 months (Table 2).

**Table 2:** Statistical analysis of ADHF outcomes according to the ejection fraction at various time points

Variable	HFrEF (N, %)		HFmrEF (N, %)		HFpEF (N, %)		*P-value*
Total	4832		2222		5798		
Outcomes							
6 months							
Primary outcomes	1314	(27.2)	533	(24.0)	1202	(20.7)	< 0.001
All-cause mortality	727	(15.1)	290	(13.1)	834	(14.4)	0.086
CV death	370	(7.7)	126	(5.7)	327	(5.6)	< 0.001
HF rehospitalization	1054	(21.8)	436	(19.6)	962	(16.6)	< 0.001
AMI	169	(3.5)	87	(3.9)	162	(2.8)	0.019
Ischemic stroke	109	(2.3)	65	(2.9)	207	(3.6)	< 0.001
1 year							
Primary outcomes	1679	(34.8)	710	(32.0)	1632	(28.2)	< 0.001
All-cause mortality	1048	(21.7)	456	(20.5)	1274	(22.0)	0.364
CV death	530	(11.0)	198	(8.9)	501	(8.6)	< 0.001
HF rehospitalization	1348	(27.9)	572	(25.7)	1297	(22.4)	< 0.001
AMI	230	(4.8)	126	(5.7)	233	(4.0)	0.009
Ischemic stroke	151	(3.1)	107	(4.8)	283	(4.9)	< 0.001
5 years							
Primary outcomes	2560	(53.0)	1120	(50.4)	2683	(46.3)	< 0.001
All-cause mortality	2215	(45.8)	1074	(48.3)	2938	(50.7)	< 0.001
CV death	1052	(21.8)	446	(20.1)	1090	(18.8)	< 0.001
HF rehospitalization	2024	(41.9)	879	(39.6)	2067	(35.7)	< 0.001
AMI	410	(8.5)	254	(11.4)	441	(7.6)	< 0.001
Ischemic stroke	318	(6.6)	219	(9.9)	606	(10.5)	< 0.001
Whole period							
Primary outcomes	2813	(58.2)	1253	(56.4)	2966	(51.2)	< 0.001
All-cause mortality	2743	(56.8)	1361	(61.3)	3700	(63.8)	< 0.001
CV death	1292	(26.7)	580	(26.1)	1386	(23.9)	< 0.001
HF rehospitalization	2203	(45.6)	972	(43.7)	2241	(38.7)	< 0.001
AMI	476	(9.9)	301	(13.6)	541	(9.3)	< 0.001
Ischemic stroke	378	(7.8)	249	(11.2)	693	(12.0)	< 0.001
Follow years, mean (SD)	3.8	(3.6)	4.1	(3.6)	3.8	(3.6)	0.002

### Predicting model for AMI in HFmrEF

We analyze the factors predicting sequent AMI in hospitalized HFmrEF in eTable 3 in the Additional File. We found history of hypertension (AHR 1.39; 95% CI 1.00-1.92; p = 0.048) dyslipidemia (AHR, 1.32; 95% CI, 1.02 to 1.70; *P* = 0.032), Gastrointestinal bleeding (AHR, 1.33; 95% CI, 1.03 to 1.71; *P* = 0.031), Peripheral arterial disease (AHR 1.53; 95% CI 1.04–2.25; p = 0.033), ischemic heart disease (AHR, 2.04; 95% CI, 1.41 to 2.95; *P* < 0.001), discharged medication of statin (AHR, 1.44; 95% CI, 1.10 to 1.87; *P* = 0.007) could be independently associated with further AMI attack. Moreover, a history of atrial fibrillation (AHR, 0.63; 95% CI, 0.46 to 0.87; *P* = 0.005) seems to be negatively correlated with incidences of AMI. We investigate LDL-C and cholesterol levels while both are relatively higher among those subjects with hypertension, dyslipidemia, gastrointestinal bleeding, peripheral arterial disease, ischemic heart disease and statin use; however, those levels in HFmrEF with AF are significantly lower than those without AF (eTable 4 in the Additional File). The change of EF% during sequent AMI (n = 141) revealed 15.6% maintained at mildly reduced, 46.8% with HFpEF and 37.6% into HFrEF (eFigure 2 in the Additional File).

## Discussion

As a result of the present study, significant differences were found between ADHF subgroups with different EF% based on the ESC/AHA definitions [4, 12]. Contrary to previous studies [3, 5-11], this investigation is based on a large-scale, long-term observation, with a secondary comparison using propensity scores. HFpEF had the highest five-year mortality (50.7%), as opposed to HFrEF, which had significantly higher in-hospital mortality (13.8%), HF rehospitalization, and CV death. In both short- and long-term studies, survival rates of survivors with ADHF were similar regardless of EF% after discharge [2, 10]. A further observation is that HFmrEF has a significantly increased rate of AMI, while HFpEF has a slightly increased rate of stroke as a result of ischemic events.

### ADHF distribution by EF% and ischemic heart burden

A number of registries maintain HFmrEF at about 10–25% of entries [10, 22]. Our results shows HFpEF as the largest subgroup (45.1%), followed by HFrEF (37.6%), and HFmrEF (17.3%), consistent with the CHARM study (17% for HFmrEF) [23], Spain study (16%) [24], ESC-HF-LT Registry of AHF (18%) [19], CHART-2 (17.1%) [5], KorAHF (16%) [8] and Swedish SwedeHF registry (21%); The disparity between HFpEF and HFrEF can be explained by (1) ADHF versus CHF or mixed; and (2) diverse racial profiling and (3) different healthcare system. In our study, HFmrEF were younger and have more patients with AF and hypertension than HFpEF. HFmrEF and HFpEF had a higher percentage of women than HFrEF. Consequently, HFmrEF has similar characteristics to HFrEF as the number of patients comorbid with ischemic heart disease (IHD) and CABG. CHART-2 Study [5] showed prevalence of IHD 38.8% in HFmrEF and 37.2% in HFrEF; whereas higher prevalence in our ADHF study (HFmrEF 67.6%, HFrEF 61.0%). A significant impact of IHD burden on ADHF could indicate the importance of ASCVD outcomes in addition to the hospitalization for HF.

### Prognosis and ADHF across the spectrum of ejection fraction

A study conducted by our group showed that in-hospital mortality was 11.7% as compared to previous studies in the MIMIC-III database (13.5%) [25] and the ARIC community study (6 ~ 12%) [26]. However, the rate is higher than that of the 3 ~ 13% in the China PEACE study [27], 4.8 ~ 7.6% in the KorAHF Registry [7, 28], 2.9 ~ 3.9% in the OPTIMIZE-HF Study [29], ESC-HF-LT Registry (Mortality in HFrEF 3.4% was higher than HFpEF 2.2% or HFmrEF 2.1%) [19, 30]. Higher proportion of diabetes (more than 40%), eGFR < 60 mL/min/1.73m^2^ (> 40%), AF burden (26.0 ~ 36.2%) in our study influence the in-hospital mortality while average diabetes (34 ~ 38%), CKD (23 ~ 26%), or AF (20 ~ 32%) in ESC-HF-LT registry [19, 30]. As a result of our study, HFrEF was significantly associated with all-cause mortality, CV death, and HF rehospitalization, whereas HFpEF exhibited higher rates of ischemic strokes, but not HFmrEF. The proportion of atrial fibrillation in HFpEF (34.4%) was higher than HFrEF (27.5%) and HFmrEF (29.6%) as SwedeHF (Swedish Heart Failure Registry) registry [31]. Although, AF was associated with similarly increased risk of death, HF hospitalization, and stroke or TIA in all ejection fraction groups [31]. An metanalysis of retrospective data also demonstrated that AF increases all-cause mortality among patients with HFpEF but not among those with HFrEF [32]. The findings of our study suggest that more comorbidities and less discharge OAC use in HFpEF may increase the risk of embolic stroke and AF burden [33]. Furthermore, strokes may have an impact on all-cause mortality by impairing function and causing fatal complications in HFpEF, while clinicians should pay great attention to ASCVD events following ADHF.

### Risk of MI and ischemic burden in HFmrEF

Consequently, HFmrEF appears potentially risky following MI compared to HFrEF and HFpEF. Even after adjusting for several patient characteristics, the correlation remains insignificant trend in our study (eTable 2). In spite of the CHART-2 Study finding that AMI, or stroke did not differ significantly between the three groups of people with CHF, the majority of the HFmrEF could transition to HFrEF as a result of IHD burden [5]. There were fewer patients in in CHART-2 Study and a shorter follow-up period than in ours, which enrolled patients with ADHF, not CHF. REPORT-HF registry reported significant IHD burden (53%) in global ADHF and 17% were admitted with new-onset IHD [2]. After discharge, ADHF with IHD had higher incidental AMI events after discharge (IHD 2% vs. non-IHD < 1%, p < 0.001). SwedeHF registry [34] showed HFmrEF has a greater proportion of IHD and AMI history as HFrEF compatible with previous studies (7 to 25% had a history of AMI) [35, 36] and recent meta-analysis [10]. As the results of the ESC HF LT Registry, the Gulf CARE registry reported acute coronary syndrome is a major precipitating factor for De novo ADHF compared to acute decompensated CHF (39% vs. 17%, p < 0.001) [3]. Ola Vedin et al. also found HFmrEF resembled HFrEF rather than HFpEF with regard to both a higher prevalence of IHD and a greater risk of new IHD events [9]. Additionally, we found that higher lipid profiles in patients with histories of IHD, discharge statin use may increase the risk of subsequent MI in patients with HFmrEF (eTable3), and this association decreased after being adjusted for other factors. According to multi-parametric cardiovascular magnetic resonance (CMR), Brown et al. reported a similar degree of fibrosis and microvascular impairment (hyperaemic myocardial blood flow) in HFpEF and HFmrEF, who also exhibit a high prevalence of occult ischemic heart disease as HFrEF [37]. Researchers also found that HFmrEF might be more sensitive to mild ischemic injury than patients with HFrEF or HFpEF based on hs-cTnT levels (HR HFrEF vs. HFmrEF vs. HFpEF: 1.71 vs. 3.76 vs. 1.87) [38, 39]. As a result of our study, 46.1% of patients with HFmrEF transitioned to HFrEF during an AMI attack, while nearly 54% of patients had LVEFs greater than 40%. According to a study done by Farr, 62% of HFmrEF patients remained at LVEF 40–50% while 24% and 33% transitioned to HFrEF and HFpEF, respectively. Since one-third of patients with HFmrEF exhibited decreased LVEF or decreased EF%, underlying coronary artery disease should be investigated [40].

Furthermore, we could confirm a correlation between IHD, particularly between new MI events and ADHF with HFmrEF, which should further reinforce research efforts into the possibly beneficial effects of revascularization in the subgroup. Ischemia type HFmrEF should be closet to HFrEF, and non-ischemic type could be closed to HFpEF focus on comorbidities prevention.

## Limitations

Retrospective data were collected from electronic records at multiple centers with hereditary bias. In addition to operator factors, echocardiographic parameters can be affected by interaction factors and the absence of multiple checks during hospitalization. In view of co-existing conditions, such as rapid AF, ADHF did not adjudicate the EF. When the patient was hospitalized, echocardiography (including an assessment of the EF) was performed in accordance with local protocol and routine (there was no core laboratory). There was a lack of information available regarding coronary artery disease and ischemic heart disease. In terms of major diagnoses, it was difficult to differentiate between AMI types. The immediate hemodynamic status, personal function status, and severity of noncardiac complications, such as sepsis, were not reported. Medical care quality and records varied from hospital to hospital, whereas validation analysis was carried out in accordance with previous reports [21]. We were unable to obtain detailed information on the clinical condition of the patient after discharge, as well as his compliance with his medications. In contrast to previous studies, our study makes use of a large cohort of ADHF and propensity scoring for variable balances, which is a unique feature.

## Conclusion

Despite appearing intermediate between HFrEF and HFpEF, our study found HFmrEF has a significant risk of subsequent myocardial infarction after ADHF. We considered vascular events as the essential outcome instead of mortality, and those results suggested that aggressive revascularization might reduce HF rehospitalization of HFmrEF patients with ischemia as well as preserve cardiac function. These findings are of importance to future research strategies on prevention and treatment of different HF types and IHD.

## Electronic supplementary material

Below is the link to the electronic supplementary material.

Additional File 1: Supplemental

## Data Availability

This study used and analyzed data sets that can be obtained upon reasonable request from the corresponding author.

## References

[1] Savarese G, Becher PM, Lund LH et al. ; Global burden of heart failure: A comprehensive and updated review of epidemiology. *Cardiovasc Res* 2022 Feb 12:cvac013. doi: 10.1093/cvr/cvac013. Epub ahead of print. PMID: 35150240. 10.1093/cvr/cvac013 35150240

[2] Tromp J, Ouwerkerk W, Cleland JGF, et al. Global differences in Burden and Treatment of Ischemic Heart Disease in Acute Heart failure: REPORT-HF. JACC Heart Fail. 2021 May;9(5):349–59. Epub 2021 Apr 7. PMID: 33839078. 10.1016/j.jchf.2020.12.015 33839078

[3] Sulaiman K, Panduranga P, Al-Zakwani I, et al. Clinical characteristics, management, and outcomes of acute heart failure patients: observations from the Gulf acute heart failure registry (Gulf CARE). Eur J Heart Fail. 2015 Apr;17(4):374–84. 10.1002/ejhf.245. Epub 2015 Mar 4. PMID: 25739882. 10.1002/ejhf.245 25739882

[4] McDonagh TA, Metra M, Adamo M et al. ; 2021 ESC Guidelines for the diagnosis and treatment of acute and chronic heart failure. *European Heart Journal* 2021; 42: 3599–3726. 10.1093/eurheartj/ehab368 34447992

[5] Tsuji K, Sakata Y, Nochioka K, et al. Characterization of heart failure patients with mid-range left ventricular ejection fraction-a report from the CHART-2 study. Eur J Heart Fail. 2017 Oct;19(10):1258–69. 10.1002/ejhf.807. Epub 2017 Mar 31. PMID: 28370829. 10.1002/ejhf.807 28370829

[6] Bhambhani V, Kizer JR, Lima JAC, et al. Predictors and outcomes of heart failure with mid-range ejection fraction. Eur J Heart Fail. 2018 Apr;20(4):651–9. 10.1002/ejhf.1091. Epub 2017 Dec 11. PMID: 29226491; PMCID: PMC5899688. 10.1002/ejhf.1091 PMC5899688 29226491

[7] Kitai T, Miyakoshi C, Morimoto T (2020). Mode of Death among japanese adults with heart failure with preserved, Midrange, and reduced ejection fraction. JAMA Netw Open May.

[8] Lee SE, Lee HY, Cho HJ, et al. Clinical characteristics and outcome of Acute Heart failure in Korea: results from the korean Acute Heart failure Registry (KorAHF). Korean Circ J. 2017 May;47(3):341–53. 10.4070/kcj.2016.0419. Epub 2017 May 25. PMID: 28567084; PMCID: PMC5449528. 10.4070/kcj.2016.0419 PMC5449528 28567084

[9] Vedin O, Lam CSP, Koh AS et al. ; Significance of ischemic heart disease in patients with heart failure and preserved, Midrange, and reduced ejection fraction: a Nationwide Cohort Study. Circ Heart Fail 2017;10(6). 10.1161/CIRCHEARTFAILURE.117.003875 28615366

[10] Liang M, Bian B, Yang Q. Characteristics and long-term prognosis of patients with reduced, mid-range, and preserved ejection fraction: a systemic review and meta-analysis. Clin Cardiol. Jan 2022;45(1):5–17. 10.1002/clc.23754. 10.1002/clc.23754 PMC8799045 35043472

[11] Shiga T, Suzuki A, Haruta S (2019). Clinical characteristics of hospitalized heart failure patients with preserved, mid-range, and reduced ejection fractions in Japan. ESC Heart Fail Jun.

[12] Heidenreich PA, Bozkurt B, Aguilar D et al. ; 2022 AHA/ACC/HFSA Guideline for the Management of Heart Failure: A Report of the American College of Cardiology/American Heart Association Joint Committee on Clinical Practice Guidelines. *J Am Coll Cardiol* 2022 May 3;79(17):e263-e421. doi: 10.1016/j.jacc.2021.12.012. Epub 2022 Apr 1. PMID: 35379503. 10.1016/j.jacc.2021.12.012 35379503

[13] Solomon SD, McMurray JJV, Anand IS (2019). Angiotensin-neprilysin inhibition in Heart failure with preserved ejection fraction. N Engl J Med.

[14] Solomon SD, McMurray JJV, Claggett B, et al. DELIVER trial committees and investigators. Dapagliflozin in Heart failure with mildly reduced or preserved ejection fraction. N Engl J Med. 2022 Sep;22(12):1089–98. 10.1056/NEJMoa2206286. Epub 2022 Aug 27. PMID: 36027570. 10.1056/NEJMoa2206286 36027570

[15] Takei M, Kohsaka S, Shiraishi Y, et al. Heart failure with midrange ejection fraction in patients admitted for Acute Decompensation: a report from the japanese Multicenter Registry. J Card Fail. 2019 Aug;25(8):666–73. 10.1016/j.cardfail.2019.05.010. Epub 2019 May 23. PMID: 31129270. 10.1016/j.cardfail.2019.05.010 31129270

[16] Rickenbacher P, Kaufmann BA, Maeder MT (2017). Heart failure with mid-range ejection fraction: a distinct clinical entity? Insights from the trial of intensified versus standard medical therapy in Elderly patients with congestive heart failure (TIME-CHF). Eur J Heart Fail.

[17] Ibrahim NE, Song Y, Cannon CP, et al. Heart failure with mid-range ejection fraction: characterization of patients from the PINNACLE Registry®. ESC Heart Fail. 2019 Aug;6(4):784–92. 10.1002/ehf2.12455. Epub 2019 Jul 3. PMID: 31268631; PMCID: PMC6676450. 10.1002/ehf2.12455 PMC6676450 31268631

[18] Uretsky BF, Thygesen K, Armstrong PW (2000). Acute coronary findings at autopsy in heart failure patients with sudden death: results from the assessment of treatment with lisinopril and survival (ATLAS) trial. Circulation.

[19] Chioncel O, Lainscak M, Seferovic PM (2017). Epidemiology and one-year outcomes in patients with chronic heart failure and preserved, mid-range and reduced ejection fraction: an analysis of the ESC Heart failure Long-Term Registry. Eur J Heart Fail.

[20] Solomon SD, Anavekar N, Skali H (2005). Candesartan in Heart failure reduction in mortality (CHARM) investigators. Influence of ejection fraction on cardiovascular outcomes in a broad spectrum of heart failure patients. Circulation.

[21] Shao SC, Chan YY, Kao Yang YH, et al. The Chang Gung Research Database-A multi-institutional electronic medical records database for real-world epidemiological studies in Taiwan. Pharmacoepidemiol Drug Saf. 2019 May;28(5):593–600. Epub 2019 Jan 16. PMID: 30648314. 10.1002/pds.4713 30648314

[22] Savarese G, Stolfo D, Sinagra G et al. ; Heart failure with mid-range or mildly reduced ejection fraction. *Nat Rev Cardiol*. 2022 Feb;19(2):100–116. doi: 10.1038/s41569-021-00605-5. Epub 2021 Sep 6. PMID: 34489589; PMCID: PMC8420965. 10.1038/s41569-021-00605-5 PMC8420965 34489589

[23] Lund LH, Claggett B, Liu J, et al. Heart failure with mid-range ejection fraction in CHARM: characteristics, outcomes and effect of candesartan across the entire ejection fraction spectrum. Eur J Heart Fail. 2018 Aug;20(8):1230–9. 10.1002/ejhf.1149. Epub 2018 Feb 12. PMID: 29431256. 10.1002/ejhf.1149 29431256

[24] Santas E, de la Espriella R, Palau P (2020). Rehospitalization burden and morbidity risk in patients with heart failure with mid-range ejection fraction. ESC Heart Fail Jun.

[25] Li F, Xin H, Zhang J (2021). Prediction model of in-hospital mortality in intensive care unit patients with heart failure: machine learning-based, retrospective analysis of the MIMIC-III database. BMJ Open Jul.

[26] Mounsey LA, Chang PP, Sueta CA et al. ; In-Hospital and Postdischarge Mortality Among Patients With Acute Decompensated Heart Failure Hospitalizations Ending on the Weekend Versus Weekday: The ARIC Study Community Surveillance. *J Am Heart Assoc* 2019 Aug 6;8(15):e011631. doi: 10.1161/JAHA.118.011631. Epub 2019 Jul 19. PMID: 31319746; PMCID: PMC6761634. 10.1161/JAHA.118.011631 PMC6761634 31319746

[27] Yu Y, Gupta A, Wu C, China PEACE, Collaborative Group. Characteristics, Management, and Outcomes of Patients Hospitalized for Heart Failure in China: The China PEACE Retrospective Heart Failure Study. *J Am Heart Assoc* 2019 Sep 3;8(17):e012884. doi: 10.1161/JAHA.119.012884. Epub 2019 Aug 21. PMID: 31431117; PMCID: PMC6755852. 10.1161/JAHA.119.012884 PMC6755852 31431117

[28] Cho JH, Choe WS, Cho HJ, et al. Comparison of characteristics and 3-Year outcomes in patients with Acute Heart failure with Preserved, mid-range, and reduced ejection fraction. Circ J. 2019 Jan;25(2):347–56. 10.1253/circj.CJ-18-0543. Epub 2018 Nov 6. PMID: 30404976. 10.1253/circj.CJ-18-0543 30404976

[29] Fonarow GC, Stough WG, Abraham WT (2007). Characteristics, treatments, and outcomes of patients with preserved systolic function hospitalized for heart failure: a report from the OPTIMIZE-HF registry. J Am Coll Cardiol.

[30] Kaplon-Cieslicka A, Benson L, Chioncel O, et al. A comprehensive characterization of acute heart failure with preserved versus mildly reduced versus reduced ejection fraction - insights from the ESC-HFA EORP heart failure Long-Term Registry. Eur J Heart Fail. Feb 2022;24(2):335–50. 10.1002/ejhf.2408. 10.1002/ejhf.2408 34962044

[31] Sartipy U, Dahlström U, Fu M, et al. Atrial fibrillation in heart failure with Preserved, mid-range, and reduced ejection fraction. JACC Heart Fail. 2017 Aug;5(8):565–74. 10.1016/j.jchf.2017.05.001. Epub 2017 Jul 12. PMID: 28711451. 10.1016/j.jchf.2017.05.001 28711451

[32] Liu G, Long M, Hu X, et al. Meta-analysis of Atrial Fibrillation and Outcomes in patients with heart failure and preserved ejection fraction. Heart Lung Circ. May 2021;30(5):698–706. 10.1016/j.hlc.2020.10.010. 10.1016/j.hlc.2020.10.010 33191141

[33] Uhm JS, Kim J, Yu HT, et al. Stroke and systemic embolism in patients with atrial fibrillation and heart failure according to heart failure type. ESC Heart Fail. 2021 Apr;8(2):1582–9. 10.1002/ehf2.13264. Epub 2021 Feb 25. PMID: 33634593; PMCID: PMC8006674. 10.1002/ehf2.13264 PMC8006674 33634593

[34] Koh AS, Tay WT, Teng THK, et al. A comprehensive population-based characterization of heart failure with mid-range ejection fraction. Eur J Heart Fail. 2017 Dec;19(12):1624–34. 10.1002/ejhf.945. Epub 2017 Sep 25. PMID: 28948683. 10.1002/ejhf.945 28948683

[35] Ito M, Wada H, Sakakura K (2019). Clinical characteristics and long-term outcomes of patients with Acute Decompensated Heart failure with mid-range ejection Fraction. Int Heart J Jul.

[36] Ueda T, Kawakami R, Nakada Y (2019). Differences in blood pressure riser pattern in patients with acute heart failure with reduced mid-range and preserved ejection fraction. ESC Heart Fail Oct.

[37] Brown LAE, Wahab A, Ikongo E et al. Cardiovascular magnetic resonance phenotyping of heart failure with mildly reduced ejection fraction. Eur Heart J Cardiovasc Imaging. 2022 Dec 19;24(1):38–45. doi: 10.1093/ehjci/jeac204. PMID: 36285884; PMCID: PMC9762938. 10.1093/ehjci/jeac204 PMC9762938 36285884

[38] Moliner P, Lupón J, Barallat J et al. ; Bio-profiling and bio-prognostication of chronic heart failure with mid-range ejection fraction. Int J Cardiol 2018 Apr 15;257:188–92. doi: 10.1016/j.ijcard.2018.01.119. Epub 2018 Jan 31. PMID: 29415801. 10.1016/j.ijcard.2018.01.119 29415801

[39] Gohar A, Chong JPC, Liew OW, et al. The prognostic value of highly sensitive cardiac troponin assays for adverse events in men and women with stable heart failure and a preserved vs. reduced ejection fraction. Eur J Heart Fail. 2017 Dec;19(12):1638–47. 10.1002/ejhf.911. Epub 2017 Aug 28. PMID: 28849609. 10.1002/ejhf.911 28849609

[40] Savarese G, Vedin O, D’Amario D (2019). Prevalence and prognostic implications of longitudinal ejection Fraction Change in Heart failure. JACC Heart Fail Apr.

