# Supplementary material for: Acute heart failure with mildly reduced ejection fraction and myocardial infarction: a multi-institutional cohort study
Source: BMC Cardiovasc Disord. 2023 May 23;23:272. doi: 10.1186/s12872-023-03286-9 (PMC10207615; doi:10.1186/s12872-023-03286-9)
Supplement: Supplementary file 1 — Additional File 1: Supplemental [file 12872_2023_3286_MOESM1_ESM.docx]

**Supplemental**

eAppendix 1. Variable Definitions

eTable 1. A comparison of baseline characteristics of ADHF patients with in-hospital death.

eTable 2. Prognostic factors for whole period outcomes in patients with left ventricular ejection fraction.

eTable 3. Cox Regression Analysis for AMI in HFmrEF patients.

eTable 4. The lipid profiles of subgroups with dyslipidemia, AF, discharge antiplatelet and discharge statins compared to those without them in patients with heart failure mildly reduced EF.

eFigure 1. The prevalence (frequency) of in-hospital mortality in ADHF with different ejection fraction.

eFigure 2. EF% transition in HFmrEF with sequent myocardial infarction.

eAppendix 1. Variable Definitions

| **Variable** | **Definition** |
| --- | --- |
| **Atrial fibrillation** | ICD9 codes of 427.31; ICD10 codes of I480-I482, I4891 |
| **Cerebrovascular disease** | ICD9 codes of 430-438; ICD10 codes of I60-I69 |
| **Chronic obstructive pulmonary disease** | ICD9 codes of 491.xx, 492.xx, 496.xx; ICD10 codes of J44 |
| **Diabetes mellitus** | ICD9 codes of 250.x1, 250.x3; ICD10 codes of E10 |
| **Dyslipidemia** | ICD9 codes of 272.xx; ICD10 codes of E77, E780, E781, E782, E783, E784, E785, E786, E881, E753, E755, E882, E756, E789, E7521, E7522, E7524, E7130, E7879, E7881, E7889, E8889, E7870 |
| **Gastrointestinal bleeding** | ICD9 codes of 530.21, 530.7, 530.82, 531.xx–534.xx, 535.xx, 537.83, 537.84, 578.xx; ICD10 codes of K22.6, K25.0, K26.0, K27.0, K28.0, K62.5, K29.01, K29.31, K29.61, K29.21, K29.31, K29.71, K29.81, K52.81 , K31.811, K31.82, K57.11, K57.01, K57.31, K57.21, K55.21, K92.0, K31.82, K56.60, K27.1, K28.1,, K29.41, K29.61, K29.91,K57.13, K57.91, K57.33,K92.1, K56.60, K26.1, K27.2, K28.2,, K29.51, K52.81, K57.81,K92.2, K25.1, K26.2, K27.3, K28.3,, K57.93, K25.2, K26.3, K27.4, K28.4, K25.3, K26.4, K27.5, K28.5, K25.4, K26.5, K27.6, K28.6, K25.5, K26.6, K27.7, K28.7, K25.6, K26.7, K27.9, K28.9, K25.7, K26.9, K25.9 |
| **Gouty arthritis** | ICD9 codes of 274.xx; ICD10 codes of M10, M1A |
| **Heart disease** | ICD9 codes of 390-392, 393-398, 410-414 and 420-429; ICD10 codes of I01-I02.0, I05-I09, I20-I25, I27 and I30-I52 |
| **Hypertension** | ICD9 codes of 401.xx, 402.xx, 403.xx, 404.xx, 405.xx; ICD10 codes of I10-I15 |
| **Intra-cranial hemorrage** | ICD9 codes of 430.xx–432.xx; ICD10 codes of I60-I62 |
| **Ischemic heart disease** | ICD9 codes of 410.xx–414.xx; ICD10 codes of I20-I25 |
| **Old ischemic stroke** | ICD9 codes of 433.xx–437.xx; ICD10 codes of I66, I65.1, I65.0, I65.8, I65.9, I63.6, I63.8, I63.9, G45.0, G45.8, G45.1, G45.2, G46.0, G46.1, G46.2, G45.9, G45.4, G46.3, G46.4, G46.5, G46.6, G46.7, G46.8, I67.0, I67.1, I67.2, I67.4, I67.5, I67.6, I67.7, I67.9, I68.0, I68.2, I68.8 |
| **Peripheral arterial disease** | ICD9 codes of 440.0x, 440.2x, 440.3x, 440.8x, 440.9x, 443.xx, 444.0x, 444.22, 444.8x, 447.8x, 447.9x; ICD10 codes of I70, I75, I73, I74, I79, I77 |
| **S/p CABG** | ICD9 codes of V45.81,414.04; ICD9 PCS code of 36.10, 36.11, 36.12, 36.13, 36.15, 36.16, 36.19, 36.20; ICD10 codes of Z951, I257, I258, T822; ICD10 PCS code of 0210083, 0210088, 0210089, 021008C, 021008W, 0210093, 0210093, 0210098, 0210099, 021009C, 021009W, 02100A3, 02100A8, 02100A9, 02100AC, 02100AW, 02100J3, 02100J8, 02100J9, 02100JC, 02100JW, 02100K3, 02100K8, 02100K9, 02100KC, 02100KW, 02100Z3, 02100Z8, 02100Z9, 02100ZC, 0210483, 0210488, 0210489, 021048C, 021048W, 0210493, 0210498, 0210499, 021049C, 021049W, 02104A3, 02104A8, 02104A9, 02104AC, 02104AW, 02104J3, 02104J8, 02104J9, 02104JC, 02104JW, 02104K3, 02104K8, 02104K9, 02104KC, 02104KW, 02104Z3, 02104Z8, 02104Z9, 02104ZC, 0211088, 0211089, 021108C, 021108W, 0211098, 0211099, 021109C, 021109W, 02110A8, 02110A9, 02110AC, 02110AW, 02110J8, 02110J9, 02110JC, 02110JW, 02110K8, 02110K9, 02110KC, 02110KW, 02110Z8, 02110Z9, 02110ZC, 0211488, 0211489, 021148C, 021148W, 0211498, 0211499, 021149C, 021149W, 02114A8, 02114A9, 02114AC, 02114AW, 02114J8, 02114J9, 02114JC, 02114JW, 02114K8, 02114K9, 02114KC, 02114KW, 02114Z8, 02114Z9, 02114ZC, 021208C, 021208W, 021209C, 021209W, 02120AC, 02120AW, 02120JC, 02120JW, 02120KC, 02120KW, 02120ZC, 021248C, 021248W, 021249C, 021249W, 02124AC, 02124AW, 02124JC, 02124JW, 02124KC, 02124KW, 02124ZC, 021308C, 021309C, 02130AC, 02130JC, 02130KC, 02130ZC, 021348C, 021349C, 02134AC, 02134JC, 02134KC, 02134ZC, 021K0Z8, 021K0Z9, 021K0ZC, 021K0ZW, 021K4Z8, 021K4Z9, 021K4ZC, 021K4ZW, 021L0Z8, 021L0Z9, 021L0ZC, 021L4Z8, 021L4Z9, 021L4ZC |
| **Valve replacement** | ICD9 codes of V43.3, V42.2; ICD9 PCS code of 35.0, 35.1, 35.2; ICD10 codes of Z954, Z952; ICD10 PCS code of 02NF3ZZ, 02NF4ZZ, 02NG3ZZ, 02NG4ZZ, 02NH3ZZ, 02NH4ZZ, 02NJ3ZZ, 02NJ4ZZ, 02NF3ZZ, 02NH3ZZ, 02RF37Z, 02RF38Z, 02RF3JZ, 02RF3KZ, X2RF332, 02RF37H, 02RF38H, 02RF3JH, 02RF3KH, 02RH37Z, 02RH38Z, 02RH3JZ, 02RH3KZ, 02RH37H, 02RH38H, 02RH3JH, 02RH3KH, 02RG37H, 02RG37Z, 02RG38H, 02RG38Z, 02RG3JH, 02RG3JZ, 02RG3KH, 02RG3KZ, 02RJ37H, 02RJ37Z, 02RJ38H, 02RJ38Z, 02RJ3JH, 02RJ3JZ, 02RJ3KH, 02RJ3KZ, 02QF0ZZ, 02QG0ZZ, 02QH0ZZ, 02QJ0ZZ, 027F04Z, 027F0DZ, 027F0ZZ, 02NF0ZZ, 027G04Z, 027G0DZ, 027G0ZZ, 02NG0ZZ, 02VG0ZZ, 027H04Z, 027H0DZ, 027H0ZZ, 02NH0ZZ, 027J04Z, 027J0DZ, 027J0ZZ, 02NJ0ZZ, 02RF07Z, 02RF08Z, 02RF0JZ, 02RF0KZ, 02RF47Z, 02RF48Z, 02RF4JZ, 02RF4KZ, 02RG07Z, 02RG08Z, 02RG0JZ, 02RG0KZ, 02RG47Z, 02RG48Z, 02RG4JZ, 02RG4KZ, 02RH07Z, 02RH08Z, 02RH0JZ, 02RH0KZ, 02RH47Z, 02RH48Z, 02RH4JZ, 02RH4KZ, 02RJ07Z, 02RJ08Z, 02RJ0JZ, 02RJ0KZ, 02RJ47Z, 02RJ48Z, 02RJ4JZ, 02RJ4KZ |
| **Venous thromboembolism** | ICD9 codes of 415.1x, 453.xx; ICD10 codes of I26, I27, T80, T81, T82, I82 |
| **Antiplatelet** | ATC code of B01AC |
| **Beta-blockers** | ATC code of C07 |
| **Calcium channel blocker** | ATC codes of C08DB01, C08DA01 |
| **Dihydropyridine calcium channel blockers** | ATC code of C08CA |
| **Diuretics** | ATC code of C03 |
| **Insulin** | ATC code of A10A |
| **Mineralocortocoid receptor antagonist** | ATC codes of C03DA01, C03DA04 |
| **Oral anticoagulant** | ATC code of B01A |
| **Oral hypoglycemic agent** | ATC codes of A10BA, A10BB, A10BG02, A10BG03, A10BH, A10BF |
| **RASi** | ATC code of C09 |
| **Statin** | ATC code of C10AA |

eTable 1. A comparison of baseline characteristics of ADHF patients with in-hospital death.

| Variable | HFrEF  (N, %) | | HFmrEF  (N, %) | | HFpEF  (N, %) | | *P-value* |
| --- | --- | --- | --- | --- | --- | --- | --- |
| Total | 773 |  | 256 |  | 677 |  |  |
| Age, mean (SD), years | 65.0 | (16.1) | 68.5 | (14.6) | 72.6 | (13.6) | <0.001 |
| Age group, years | 71.3 | (15.6) | 74.4 | (13.2) | 77.4 | (13.2) | <0.001 |
| 18–64 | 226 | (29.2) | 67 | (26.2) | 108 | (16.0) |  |
| 65–74 | 176 | (22.8) | 45 | (17.6) | 115 | (17.0) |  |
| ≥ 75 | 371 | (48.0) | 144 | (56.3) | 454 | (67.1) |  |
| Sex |  |  |  |  |  |  | <0.001 |
| Male | 518 | (67.0) | 151 | (59.0) | 274 | (40.5) |  |
| Female | 255 | (33.0) | 105 | (41.0) | 403 | (59.5) |  |
| HF diagnosis year |  |  |  |  |  |  | 0.210 |
| Before 2016 | 567 | (73.4) | 200 | (78.1) | 491 | (72.5) |  |
| After 2016 | 206 | (26.7) | 56 | (21.9) | 186 | (27.5) |  |
| Hospital level |  |  |  |  |  |  | 0.023 |
| Medical center | 502 | (64.9) | 190 | (74.2) | 451 | (66.6) |  |
| Non-medical center | 271 | (35.1) | 66 | (25.8) | 226 | (33.4) |  |
| Comorbidities |  |  |  |  |  |  |  |
| Hypertension | 458 | (59.3) | 173 | (67.6) | 458 | (67.7) | 0.002 |
| Diabetes mellitus | 355 | (45.9) | 119 | (46.5) | 292 | (43.1) | 0.486 |
| Dyslipidemia | 169 | (21.9) | 61 | (23.8) | 136 | (20.1) | 0.431 |
| Atrial fibrillation | 201 | (26.0) | 75 | (29.3) | 245 | (36.2) | <0.001 |
| Peripheral arterial disease | 60 | (7.8) | 19 | (7.4) | 46 | (6.8) | 0.778 |
| VTE | 35 | (4.5) | 15 | (5.9) | 53 | (7.8) | <0.001 |
| COPD | 134 | (17.3) | 49 | (19.1) | 169 | (25.0) | 0.001 |
| Gouty arthritis | 92 | (11.9) | 35 | (13.7) | 64 | (9.5) | 0.133 |
| Gastrointestinal bleeding | 203 | (26.3) | 79 | (30.9) | 216 | (31.9) | 0.051 |
| Intra-cranial hemorrhage | 28 | (3.6) | 5 | (2.0) | 15 | (2.2) | 0.180 |
| Ischemic heart disease | 507 | (65.6) | 174 | (68.0) | 294 | (43.4) | <0.001 |
| Old ischemic stroke | 127 | (16.4) | 47 | (18.4) | 124 | (18.3) | <0.001 |
| S/p CABG | 61 | (7.9) | 15 | (5.9) | 17 | (2.5) | <0.001 |
| Valve replacement | 32 | (4.1) | 5 | (2.0) | 29 | (4.3) | 0.224 |
| eGFR, mL/min/1.73m^2^ | 529 |  | 168 |  | 447 |  | <0.001 |
| ≥60 | 370 | (69.9) | 110 | (65.5) | 319 | (71.4) | 0.365 |
| 30–59 | - | - | - | - | - | - |  |
| 16–30 | - | - | - | - | - | - |  |
| Dialysis | 159 | (30.1) | 58 | (34.5) | 128 | (28.6) |  |
| Hospitalized Intervention |  |  |  |  |  |  |  |
| Intubation/ventilation | 175 | (22.6) | 60 | (23.4) | 111 | (16.4) | 0.005 |
| Intensive Care Unit stay | 476 | (61.6) | 157 | (61.3) | 356 | (52.6) | 0.001 |
| Intensive Care Unit stay, mean (SD), days | 18.6 | (24.2) | 20.2 | (21.8) | 19.2 | (22.6) | 0.758 |
| NIPPV | 38 | (4.9) | 11 | (4.3) | 33 | (4.9) | 0.917 |
| CPCR | 18 | (2.3) | 5 | (2.0) | 3 | (0.4) | 0.012 |
| Cardioversion | 12 | (1.6) | 2 | (0.8) | 4 | (0.6) | 0.182 |
| Blood transfusion | 68 | (8.8) | 31 | (12.1) | 71 | (10.5) | 0.260 |
| Hemodialysis | 23 | (3.0) | 11 | (4.3) | 20 | (3.0) | 0.533 |
| Coronary PCI with stenting | 8 | (1.0) | 1 | (0.4) | 3 | (0.4) | 0.328 |
| Abbreviations: ALT, Alanine aminotransferase; AST, Aspartate aminotransferase; BNP, B-type natriuretic peptide; CCB, calcium channel blockers; COPD, Chronic obstructive pulmonary disease; CPCR, Cardio-Pulmonary-Cerebral-Resuscitation; DCCB, dihydropyridine calcium channel blockers; eGFR, estimated Glomerular filtration rate; HFmrEF, heart failure with mid-range ejection fraction; HFpEF, heart failure with preserved ejection fraction; HFrEF, heart failure with reduced ejection fraction; MRA, mineralocortocoid receptor antagonist; NIPPV, Noninvasive positive pressure ventilation; NT-ProBNP, N-terminal Pro-Brain Natriuretic Peptide; OAC, oral anticoagulants; OHA, oral hypoglycemic agent; PCI, percutaneous coronary intervention; RASi, angiotensin converting enzyme inhibitors, angiotensin receptor blocker, or angiotensin receptor–neprilysin inhibitor; S/p CABG, status post coronary artery bypass graft; VTE, Venous thromboembolism. | | | | | | | |

eTable 2 Prognostic factors for whole period outcomes in patients with left ventricular ejection fraction

| **Outcomes** | **Crude** | | **Adjusted^a^** | |
| --- | --- | --- | --- | --- |
|  | HR (95% CI) | *P-value* | HR (95% CI) | *P-value* |
| **Whole period** |  |  |  |  |
| Primary outcomes |  |  |  |  |
| HFrEF | 1.29 (1.22-1.35) | <0.001 | 1.55 (1.42-1.69) | <0.001 |
| HFmrEF | 1.15 (1.07-1.23) | <0.001 | 1.27 (1.14-1.41) | <0.001 |
| HFpEF | Ref. |  | Ref. |  |
| All-cause mortality |  |  |  |  |
| HFrEF | 0.90 (0.85-0.94) | <0.001 | 1.24 (1.18-1.31) | <0.001 |
| HFmrEF | 0.90 (0.85-0.96) | 0.001 | 1.11 (1.04-1.18) | 0.002 |
| HFpEF | Ref. |  | Ref. |  |
| CV death |  |  |  |  |
| HFrEF | 1.20 (1.12-1.30) | <0.001 | 1.35 (1.24-1.47) | <0.001 |
| HFmrEF | 1.09 (0.99-1.20) | 0.091 | 1.17 (1.06-1.30) | 0.002 |
| HFpEF | Ref. |  | Ref. |  |
| HF readmission |  |  |  |  |
| HFrEF | 1.29 (1.22-1.37) | <0.001 | 1.34 (1.25-1.43) | <0.001 |
| HFmrEF | 1.16 (1.08-1.25) | <0.001 | 1.17 (1.09-1.27) | <0.001 |
| HFpEF | Ref. |  | Ref. |  |
| AMI |  |  |  |  |
| HFrEF | 1.10 (0.97-1.24) | 0.144 | 0.99 (0.87-1.13) | 0.881 |
| HFmrEF | 1.47 (1.27-1.69) | <0.001 | 1.15 (0.99-1.32) | 0.067 |
| HFpEF | Ref. |  | Ref. |  |
| Ischemic stroke |  |  |  |  |
| HFrEF | 0.66 (0.58-0.74) | <0.001 | 0.72 (0.63-0.82) | <0.001 |
| HFmrEF | 0.92 (0.80-1.06) | 0.250 | 0.93 (0.80-1.08) | 0.348 |
| HFpEF | Ref. |  | Ref. |  |
| ^a^Models were adjusted by left ventricular ejection fraction, sex, age group, hypertension, diabetes mellitus, dyslipidemia, atrial fibrillation, peripheral arterial disease, chronic obstructive pulmonary disease, gouty arthritis, gastrointestinal bleeding, intra-cranial hemorrhage, ischemic heart disease, old ischemic stroke, S/p CABG, valve replacement, intunation/ventilation, ICU stay, discharged antiplatelet, discharged OAC, discharged RASi, discharged beta-blockers, discharged DCCB, discharged diuretics and discharged statin. | | | | |

eTable 3. Cox Regression Analysis for AMI in HFmrEF patients.

| Variable | **Crude** | | **Adjusted^a^** | |
| --- | --- | --- | --- | --- |
|  | HR (95% CI) | *P-value* | HR (95% CI) | *P-value* |
| Age group |  |  |  |  |
| 18 – 64 years | Ref. |  | Ref. |  |
| 65 – 74 years | 1.06 (0.80-1.41) | 0.694 | 0.98 (0.73-1.32) | 0.877 |
| ≥ 75 years | 0.93 (0.71-1.20) | 0.566 | 0.98 (0.73-1.31) | 0.872 |
| Sex |  |  |  |  |
| Male | 1.11 (0.88-1.41) | 0.365 | 0.98 (0.76-1.27) | 0.881 |
| Female | Ref. |  | Ref. |  |
| Comorbidities |  |  |  |  |
| Hypertension | 1.85 (1.37-2.49) | <0.001 | 1.39 (1.00-1.92) | 0.048 |
| Diabetes mellitus | 1.95 (1.55-2.47) | <0.001 | 1.18 (0.91-1.53) | 0.202 |
| Dyslipidemia | 2.25 (1.79-2.82) | <0.001 | 1.32 (1.02-1.70) | 0.032 |
| Atrial fibrillation | 0.47 (0.35-0.64) | <0.001 | 0.63 (0.46-0.87) | 0.005 |
| Peripheral arterial disease | 2.07 (1.43-3.00) | <0.001 | 1.53 (1.04-2.25) | 0.033 |
| Venous thromboembolism | 0.58 (0.26-1.30) | 0.186 | 0.63 (0.27-1.47) | 0.287 |
| COPD | 0.86 (0.64-1.17) | 0.346 | 0.95 (0.69-1.32) | 0.756 |
| Gouty arthritis | 1.00 (0.71-1.40) | 0.992 | 0.85 (0.60-1.21) | 0.378 |
| Gastrointestinal bleeding | 1.45 (1.13-1.84) | 0.003 | 1.33 (1.03-1.71) | 0.031 |
| Intra-cranial hemorrhage | 1.78 (0.94-3.39) | 0.078 | 1.58 (0.83-2.99) | 0.163 |
| Ischemic heart disease | 3.51 (2.49-4.94) | <0.001 | 2.04 (1.41-2.95) | <0.001 |
| Old ischemic stroke | 1.52 (1.15-2.01) | 0.003 | 1.28 (0.95-1.72) | 0.105 |
| S/p CABG | 1.65 (1.13-2.40) | 0.010 | 1.09 (0.74-1.62) | 0.671 |
| Medications at the time of discharge |  |  |  |  |
| Antiplatelet | 2.61 (1.95-3.48) | <0.001 | 1.84 (0.95-3.56) | 0.069 |
| OAC | 2.03 (1.48-2.77) | <0.001 | 0.76 (0.38-1.51) | 0.427 |
| RASi | 1.09 (0.85-1.40) | 0.488 | 0.98 (0.75-1.27) | 0.850 |
| ß-blockers | 1.37 (1.08-1.73) | 0.008 | 1.11 (0.87-1.41) | 0.411 |
| MRA | 0.77 (0.54-1.11) | 0.164 | 0.86 (0.59-1.26) | 0.439 |
| Statin | 2.33 (1.86-2.92) | <0.001 | 1.44 (1.10-1.87) | 0.007 |
| ^a^Models were adjusted by sex, age group, hypertension, diabetes mellitus, dyslipidemia, atrial fibrillation, peripheral arterial disease, venous thromboembolism, COPD , gouty arthritis, gastrointestinal bleeding, intra-cranial hemorrhage, ischemic heart disease, old ischemic stroke, S/p CABG, discharged antiplatelet, discharged OAC, discharged RASi, discharged ß-blockers, discharged MRA and discharged statin. | | | | |

eTable 4. The lipid profiles of subgroups with dyslipidemia, AF, discharge antiplatelet and discharge statins compared to those without them in patients with heart failure mildly reduced EF.

| Variable | LDL-C | | | Cholesterol | | |
| --- | --- | --- | --- | --- | --- | --- |
|  | N | mean ± SD | *P-value* | N | mean ± SD | *P-value* |
| Dyslipidemia |  |  | <0.001 |  |  | <0.001 |
| Yes (N=806) | 631 | 113.5 ±42.9 |  | 681 | 182.4 ±51.5 |  |
| No (N=1416) | 841 | 97.1 ±34.0 |  | 934 | 160.0 ±39.4 |  |
| Atrial fibrillation |  |  | <0.001 |  |  | <0.001 |
| Yes (N=657) | 406 | 90.7 ±30.6 |  | 434 | 154.1 ±36.5 |  |
| No (N=1565) | 1066 | 109.3 ±40.5 |  | 1181 | 175.1 ±48.1 |  |
| Discharge Antiplatelet |  |  | 0.010 |  |  | <0.001 |
| Yes (N=1397) | 1034 | 105.8 ±39.6 |  | 1114 | 171.9 ±47.4 |  |
| No (N=825) | 438 | 100.1 ±36.9 |  | 501 | 163.9 ±43.0 |  |
| Discharge Statin |  |  | <0.001 |  |  | <0.001 |
| Yes (N=693) | 610 | 114.7 ±43.4 |  | 636 | 182.3 ±52.0 |  |
| No (N=1529) | 862 | 96.6 ±33.4 |  | 979 | 161.1 ±39.9 |  |


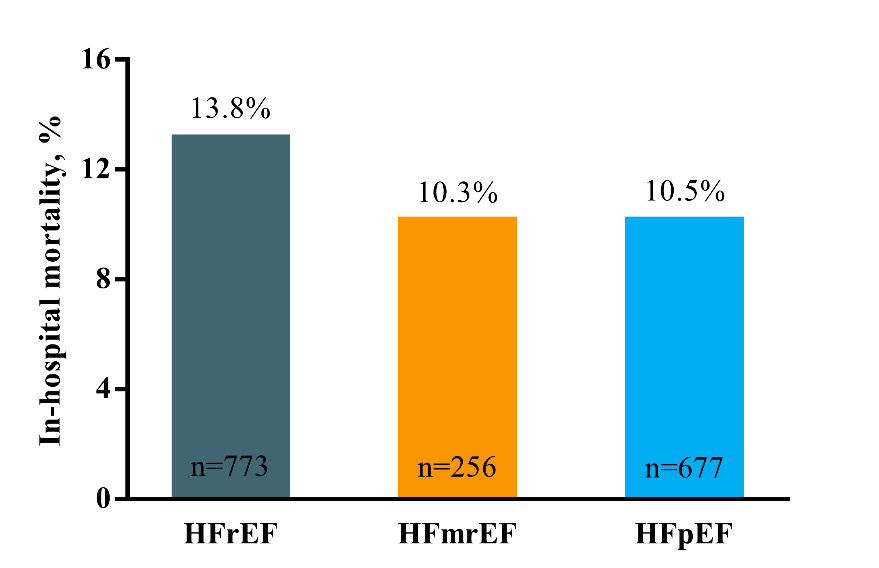


eFigure 1. The prevalence (frequency) of in-hospital mortality in ADHF with different ejection fraction


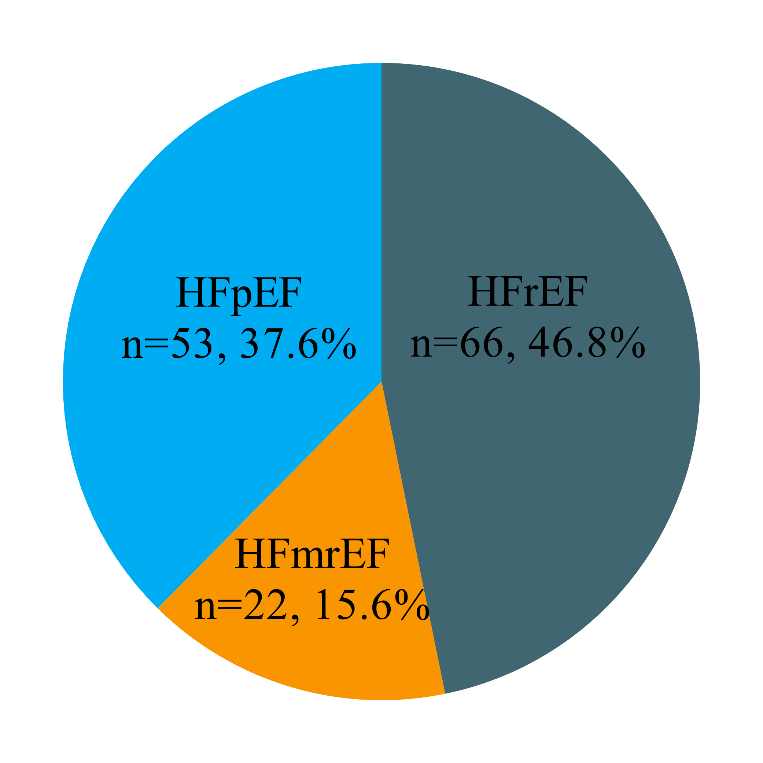


eFigure 2. EF% transition in HFmrEF with sequent myocardial infarction
